# Disposable Amperometric Immunosensor for the Determination of Human P53 Protein in Cell Lysates Using Magnetic Micro-Carriers

**DOI:** 10.3390/bios6040056

**Published:** 2016-11-21

**Authors:** María Pedrero, F. Javier Manuel de Villena, Cristina Muñoz-San Martín, Susana Campuzano, María Garranzo-Asensio, Rodrigo Barderas, José M. Pingarrón

**Affiliations:** 1 Departamento de Química Analítica, Facultad de CC. Químicas, Universidad Complutense de Madrid, E-28040 Madrid, Spain; mpedrero@quim.ucm.es (M.P.); villena@quim.ucm.es (F.J.M.d.V.); cmunoz04@ucm.es (C.M.-S.M.); susanacr@quim.ucm.es (S.C.); 2 Departamento de Bioquímica y Biología Molecular, Facultad de CC. Químicas, Universidad Complutense de Madrid, E-28040 Madrid, Spain; mgarranzo@hotmail.com (M.G.-A.); rbarderas@quim.ucm.es (R.B.)

**Keywords:** human p53, magnetic microcarriers, screen-printed electrodes, amperometric immunosensor, cell lysates

## Abstract

An amperometric magnetoimmunosensor for the determination of human p53 protein is described in this work using a sandwich configuration involving the covalent immobilization of a specific capture antibody onto activated carboxylic-modified magnetic beads (HOOC-MBs) and incubation of the modified MBs with a mixture of the target protein and horseradish peroxidase-labeled antibody (HRP-anti-p53). The resulting modified MBs are captured by a magnet placed under the surface of a disposable carbon screen-printed electrode (SPCE) and the amperometric responses are measured at −0.20 V (vs. an Ag pseudo-reference electrode), upon addition of hydroquinone (HQ) as a redox mediator and H_2_O_2_ as the enzyme substrate. The magnetoimmunosensing platform was successfully applied for the detection of p53 protein in different cell lysates without any matrix effect after a simple sample dilution. The results correlated accurately with those provided by a commercial ELISA kit, thus confirming the immunosensor as an attractive alternative for rapid and simple determination of this protein using portable and affordable instrumentation.

## 1. Introduction

Following heart disease, cancer is one of the main occurring diseases worldwide, the number of new cases is expected to rise by about 70% in the next two decades [1,2,3]. Tumor biomarkers are substances or processes associated with cancer whose levels in biological fluids or body tissues can provide essential information for clinical cancer screening and early cancer detection [4]. To reduce and control cancer, implementation of evidence-based strategies for its prevention, early detection and management of patients is needed. Although nowadays quite a number of cancer-related proteins and biomarkers have been identified, to be clinically useful simple and reproducible analysis procedures need to be developed for routine use. The implementation of simple, accurate, and low-cost detection systems for clinical biomarkers ideated to be used at home or in the field for personal healthcare and diagnostic is, in fact, one of the main objectives in the clinical research field. P53 is a DNA binding protein known in cancer biology as a critical tumor suppressor and transcription factor, proposed as the master regulator of cell fate and regarded as “the guardian of the genome” [5,6,7]. It is considered to play a crucial role in the regulation of the cell cycle, DNA repair, and programmed cell death, inhibiting the growth of tumor cells through eliciting either cell-cycle arrest or apoptosis. In cells with disruption in cell proliferation, p53 is activated and bound to the specific DNA sequences and, as a result, the uncontrolled cell growth is stopped or the damaged DNA is eliminated [4]. Loss of p53 function results in induction of tumors and gene mutation, which is caused by the conformational changes in the p53 protein structure [3,4,7,8]. Mutations of the p53 gene are the most common genetic alterations in human cancers. These mutations lead to the accumulation of the mutated p53 protein, which then may fail to bind the consensus double-stranded DNA and lose the binding activity to its downstream genes [2,9]. When tumor cells die and disintegrate, p53 protein is released and enters into the circulation. A significant increase in the serum p53 protein level in a variety of human cancers has been reported [3]. In fact, more than 50% of human cancers are related to the mutated p53, resulting in the increase of clinical possibilities for both diagnosis and treatment. Consequently, the accurate determination of p53 protein has become a great method for early diagnosis and prognosis of cancers.

Different methodologies have been described for the determination of thre p53 protein, some of them based on voluminous non-portable instrumentation that also require highly-skilled personnel, such as high-performance liquid chromatography coupled to matrix-assisted laser desorption/ionization time-of-flight mass spectrometry [10], or being time-consuming, such as the popular enzyme-linked immunosorbent assay (ELISA) [11]. P53 protein has also been determined by surface plasmon resonance (SPR) [5], impedance measurements [12,13], or electrochemiluminiscence [3]. While SPR responses are affected by film uniformity and surface conditions, the electrochemiluminiscence assay involves complex nanostructures, such as streptavidin-modified gold nanoparticles (AuNPs)/thiolated graphene oxide nanocomposite as an electrode modifier where the biotinylated capture antibody was immobilized, and a Ru-silica@Au nanocomposite-labeled secondary antibody to amplify the measured signals. 

Electrochemical immunosensors have contributed extensively to the implementation of new methodologies requiring simple, portable, and low-cost instrumentation ideally suited for future point-of-care (POC) testing systems. In fact, a few electrochemical immunosensors have been reported for the detection of p53 protein. Xie et al. [9] reported a graphene-based immunosensor for the electrochemical quantification of phosphorylated p53 on serine 15 where the biotinylated detector antibody was labeled with streptavidin-peroxidase and the electrocatalytic response to the reduction of hydrogen peroxide in the presence of thionine by differential pulse voltammetry (DPV) was used to monitor the immunocomplex formation. Additionally, Du et al. [7] reported the detection of phosphorylated p53 at Ser392 using a secondary antibody modified with graphene oxide as a nanocarrier of horseradish peroxidase (HRP) and AuNPs-modified screen-printed carbon electrodes (SPCEs) modified with a self-assembled monolayer of *N*-hydroxysuccinimide activated hexa(ethylene glycol) undecane thiol for attachment of the capture antibodies. In this case, square wave voltammetry (SWV) was used for monitoring the immunocomplex formation based on the thionine/hydrogen peroxide system. The same group proposed a multiplexed electrochemical immunoassay of phosphorylated p53 proteins using gold nanorods (AuNRs) as nanocarriers for co-immobilization of HRP and the detector antibody, and gold working electrodes modified with NHS to incubate the capture antibody [8]. Carbon nanospheres and apoferritin protein cage (iron storage protein) nanoparticles were used by Chen et al. [14] for signal amplification in a sandwich immunocomplex for phosphorylated p53 at serine 15. Magnetic particles (MBs) were used to immobilize the capture antibodies and the lead ions liberated from the Pb_3_(PO_4_)_2_ core within the apoferritin shell were detected by stripping SWV at an in situ plated Bi film formed on a SPCE. Very recently, Afsharan et al. [4] have proposed the detection of p53 protein sandwiched between a biotinylated capture antibody immobilized on the surface of a GCE using thiolated graphene oxide/streptavidin-AuNPs and a secondary antibody labeled with HRP. The electrocatalytic reduction of thionine in the presence of hydrogen peroxide was, again, used in the monitoring of the immunocomplex formation. Although, as it will be commented later, all of these approaches showed good sensitivities, they make use of complex nanostructures which need laborious and time-consuming procedures to be implemented.

In this paper, an amperometric immunosensor for the determination of p53 protein in cell lysates is described making use of the well-known excellent characteristics of magnetic microparticles for the selective, rapid, easy, and efficient capture of biomolecules from complex samples. A simple sandwich configuration is proposed based on the use of an HRP-labeled detector antibody and the hydroquinone/hydrogen peroxide (HQ/H_2_O_2_) system to detect the captured immunocomplexes on SPCEs. 

## 2. Materials and Methods

### 2.1. Apparatus and Electrodes

Amperometric measurements were performed with a CHI812B potentiostat (CH Instruments, Austin, TX, USA) controlled by software CHI812B. SPCEs (DRP-110, DropSens, Llanera (Asturias), Spain), consisting of a 4-mm diameter carbon working electrode, a carbon counter electrode, and a Ag pseudo-reference electrode, were employed as transducers and a specific cable connector (DRP-CAC also from DropSens, S.L.) acted as an interface between the SPCEs and the potentiostat. All measurements were carried out at room temperature.

A Vortex Bunsen AGT-9 (Velp Scientifica, Usmate, MB, Italy) for the homogenization of the solutions, a Thermomixer MT100 constant temperature incubator shaker (Universal Labortechnik, Leipzig, Germany), a magnetic separator DynaMag™-2 Magnet (ThermoFisher Scientific, Waltham, MA, USA) and a Magellan V 7.1 (TECAN, Männedorf, Switzerland) ELISA plate reader were also employed. Capture of the modified MBs onto the SPCE surface was controlled by placing it in a homemade Teflon casing with an embedded neodymium magnet (AIMAN GZ) [15]. 

### 2.2. Reagents and Solutions

All reagents were of the highest available grade. Carboxylic acid-modified MBs (HOOC-MBs, 2.7 µm·Ø, 10 mg·mL^−1^, Dynabeads^TM^ M-270 Carboxylic Acid, Cat. No. 14305D) were purchased from Invitrogen-Thermo Fisher (Waltham, MA, USA). 

N-terminal Glutathione S-Transferase (GST)-tagged recombinant full length human p53 protein, expressed in *Escherichia coli* (EMD Millipore Corporation, Darmstadt, Germany, Catalog #14-865), mouse anti-p53 monoclonal antibody (used as capture antibody, AbC, from BD Transduction Laboratories™, Franklin Lakes, NJ, USA, ref. 610183), and mouse anti-p53 monoclonal antibody conjugated to HRP (used as detector antibody, AbD, from NOVUS BIOLOGICALS, Abingdon Oxon, UK, NBP2-34434H) were employed. A human total p53 DuoSet IC ELISA (from R and D Systems, Inc., Minnneapolis, MN, USA, Catalog Number DYC1043) was used to perform the ELISA assay. 

Sodium chloride, potassium chloride, sodium di-hydrogen phosphate, di-sodium hydrogen phosphate, sulfuric acid, and Tris-hydroxymethyl aminomethane-HCl (Tris-HCl) were purchased from Scharlab (Barcelona, Spain). *N*-(3-dimethylaminopropyl)-*N′*-ethylcarbodiimide (EDC) was purchased from Acros Organics (Thermo Fisher, Waltham, MA, USA). *N*-hydroxysulfosuccinimide (Sulfo-NHS), ethanolamine, HQ, Tween^®^ 20 and hydrogen peroxide (30%, w/v), sodium monohydrogen carbonate (≥99.7%), and disodium carbonate (≥99.0%) were purchased from Sigma-Aldrich (St. Louis, MO, USA). Ethylenediaminetetraacetic acid (EDTA, Merck, Madrid, Spain), 2-(*N*-morpholino)ethanesulfonic acid (MES, Gerbu Biotechnik, Heidelberg, Germany, GmbH), and a commercial blocker casein solution (a ready-to-use, PBS solution of 1% w/v purified casein, Thermo Scientific, Thermo Fisher, Waltham, MA, USA) were used. Human hemoglobin (Sigma-Aldrich, St. Louis, MO, USA, H7379), IgG from human serum (Sigma-Aldrich, St. Louis, MO, USA, I2511), bovine serum albumin (BSA Type VH, Gerbu Biotechnik, Heidelberg, Germany, GmbH), recombinant human ErbB2 protein (Sino Biological Inc., North Wales, PA, USA, SEKA10004), recombinant human progesterone receptor (PR, R&D Systems Inc., Minneapolis, MN, USA, in Human Total Progesterone R/NR3C3 DuoSet IC ELISA, ref. DYC5415), recombinant human estrogen receptor α (ERα, R and D Systems Inc., Minneapolis, MN, USA in Human Total ER alpha/NR3A1 DuoSet IC ELISA, ref. DYC5715) and recombinant human TNFα protein (in TNF-alpha EIA Kit, ELISA, RUO, 12 × 8 wells, Beckman Coulter, Alcobendas, Madrid, Spain, ref. IM1121) were also used for the selectivity study. 

The following solutions, prepared with water from a Millipore Milli-Q purification system (18.2 MΩ·cm), were employed: 0.05 M phosphate buffer, pH 6.0; 0.1 M phosphate buffer, pH 8.0; phosphate-buffered saline (PBS) consisting of 0.01 M phosphate buffer solution containing 137 mM NaCl and 2.7 mM KCl, pH 7.5; 0.025 M MES buffer, pH 5.0; 0.01 M sodium phosphate buffer consisting of PBS with 0.05% Tween^®^ 20 (pH 7.5, PBST) and 0.1 M Tris-HCl buffer, pH 7.2.

Activation of the HOOC-MBs and blocking steps were carried out using an EDC/sulfo-NHS mixture solution (50 mg·mL^−1^ each in MES buffer, pH 5.0) and a 1 M ethanolamine solution (prepared in 0.1 M phosphate buffer solution, pH 8.0), respectively.

### 2.3. MBs Functionalization and Sandwich Immunoassay

Unless otherwise stated, the MBs were placed in the magnetic separator and concentrated for 3 min before removing the supernatant after all of the involved steps. All incubation and washing steps were performed at 25 °C by incubating the MBs in 25 and 50 µL of the corresponding solution, respectively.

The functionalization of MBs was performed as follows: a 3 µL-aliquot of HOOC-MBs was transferred into a 1.5 mL Eppendorf^®^ tube and washed twice for 10 min with MES buffer at 25 °C and under continuous stirring (950 rpm). Surface MBs carboxyl groups were activated by incubation in 25 µL of a freshly EDC/sulfo-NHS solution (50 mg·mL^−1^ each prepared in MES buffer, pH 5.0) during 35 min at 25 °C (950 rpm). Subsequently, MBs were washed twice with MES buffer and incubated for 30 min (950 rpm) in 25 µL of 5 µg·mL^−1^ AbC solution (prepared in MES buffer). Thereafter, the AbC-MBs were washed twice with MES buffer (pH 5.0) and incubated in a 1 M ethanolamine solution (prepared in 0.1 M phosphate buffer, pH 8.0) for 15 min at 950 rpm in order to block the remaining unreacted groups on the surface of the activated MBs. The MBs were washed by incubating 15 min with the blocker casein solution (25 °C, 950 rpm) and stored at 4 °C in 50 µL of filtered PBS until use.

The sandwich immunoassay was carried out by re-suspending the AbC-MBs in 25 µL of a mixture solution containing a variable concentration of the target protein (or the sample to be analyzed) and 5000 diluted HRP-AbD (prepared in blocker casein solution) and incubating for 30 min (25 °C, 950 rpm) in order to form the sandwich immunocomplexes (AbC-p53-HRP-AbD) onto the MBs surface. The modified MBs were then washed twice with PBST solution and re-suspended in 50 µL 0.05 M phosphate buffer (pH 6.0) to perform the amperometric measurements.

### 2.4. Amperometric Detection

Amperometric measurements were carried out by pipetting the 50 µL of the modified MB suspension on the working electrode surface of the SPCE, which was previously positioned horizontally on a homemade Teflon casing with a neodymium magnet encapsulated. Then, the SPCE/magnet holding block ensemble was immersed into an electrochemical cell containing 10 mL 0.05 M phosphate buffer of pH 6.0 and 1.0 mM HQ (prepared just before performing the electrochemical measurement). Amperometry in stirred solutions was made by applying a detection potential of −0.20 V (vs. the Ag pseudo-reference electrode). After baseline stabilization for 60 s, 50 μL of a 0.1 M H_2_O_2_ solution were added to the electrochemical cell and the current recorded until the steady-state current was reached (approx. 100 s). The amperometric signals given through the manuscript corresponded to the difference between the steady-state and the background currents.

### 2.5. Cell Culture and Lysate Production

KM12SM and KM12C cell lines obtained from I. Fidler’s laboratory (MD Anderson Cancer Center, Houston, TX, USA), and SW480, SW620, MCF-7, MDA-MB-436, and BxPC3 cell lines obtained from the American Type Culture Collection (ATCC, Teddington, Middlesex TW11 0LY, UK) cell repository, were grown according to established protocols in DMEM (Dulbecco’s modified Eagle’s medium), supplemented with 10% fetal bovine serum, penicillin and streptomycin, and 2.5 mM l-glutamine (GIBCO-Invitrogen, Carlsbad, CA, USA) supplemented with recommended nutrients. For cell lysis, cells were washed with cold PBS, incubated for 5 min with PBS 4 mM EDTA to detach them from the plates, centrifuged at 1200 rpm to remove PBS 4 mM EDTA, and subjected to the addition of 1 mL cold lysis buffer (25 mM Tris-HCl pH 7.6, 150 mM NaCl, 1% NP-40, 1% sodium deoxycholate, 0.1% SDS) supplemented with 1X protease inhibitor cocktail, 1 mM phenylmethylsulfonyl fluoride, and 1 mM activated sodium orthovanadate. Then, the cells were incubated on ice for 10 min, passed through a 25 gauge needle attached on a 1 mL syringe 10 times and transferred to a microcentrifuge tube. The cell lysate was then clarified by centrifuging at 13,200 rpm at 4 °C for 15 min. Total protein concentration was determined using a BCA protein assay kit (Pierce, Rockford, IL, USA) and the lysates stored at −80 °C until further use.

### 2.6. Sodium Dodecyl Sulfate-Polyacrylamide (SDS-PAGE) Immunodetection Analysis

Cancer cell lysates (10 µg of each protein extract) were analyzed by 10% SDS-PAGE with Coomassie Blue R-250 staining (Sigma-Aldrich, St. Louis, MO, USA). 

For immunodetection, 10 µg of each protein extract were run in parallel using 10% SDS-PAGE. Then, proteins were transferred to nitrocellulose membranes (Hybond-C extra) using semi-dry transfer (Bio-Rad, Hercules, CA, USA) [16,17]. After blocking, membranes were incubated at optimized dilutions with alternatively antiPR monoclonal antibody (R&D Systems, Minneapolis, MN, USA) or anti-tubulin monoclonal antibody (Sigma) as loading control followed by incubation with SAv-HRP (R&D Systems, Minneapolis, MN, USA) at 1:1000 dilution or HRP-anti-mouse IgG (Pierce, Thermo Fisher Scientific, Waltham, MA, USA) at 1:5000 dilution, respectively. Specific reactive proteins were visualized with SuperSignal West Pico Maximum Sensitivity Substrate (Pierce, Thermo Fisher Scientific, Waltham, MA, USA).

### 2.7. Analysis of Real Samples

The developed immunosensor was employed for the determination of the target protein in cell lysates containing different endogenous concentrations of p53 protein. The antiPR-MBs were re-suspended in 25 µL of blocker casein solution supplemented with 2.0 µg of cell lysates and 5000 diluted HRP-AbD. The protocols described in Section 2.3 and Section 2.4 were followed to form the sandwich immunocomplexes and perform the amperometric detection. Since no matrix effect was apparent under the mentioned conditions, determination of p53 in cell lysates was carried out by interpolation of the measured amperometric signals into the calibration graph constructed with standards. The same lysate samples (1.0 µg, samples SW480 and MDA-MB-436, and 2.5 µg of each other sample) were also analyzed by an ELISA method involving the use of the same immunoreagents. 

## 3. Results and Discussion

**Figure 1:**
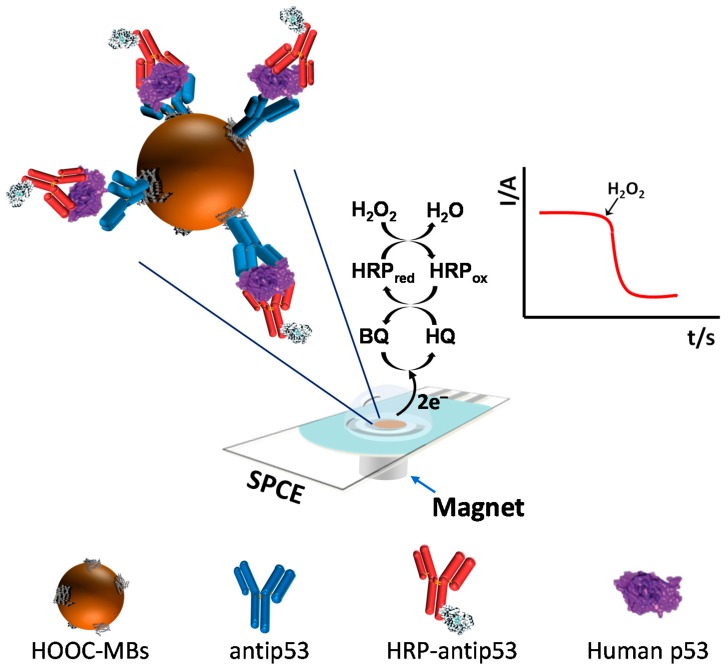
Schematic illustration of the fundamentals of the magneto-actuated amperometric sandwich immunosensor developed for human p53 determination (relative sizes of the components are not drawn to scale).

The fundamentals of the immunosensor design and the involved electrochemical transduction are schematically displayed in Figure 1. Using this strategy, all immunoreactions occurred on the MB surface while the SPCE acted just as the electrochemical transducer. Briefly, the specific capture antibody (AbC) was covalently immobilized onto HOOC-MBs activated previously with an EDC/sulfo-NHS solution. After a blocking protocol with ethanolamine of the unreacted activated groups on the MBs and washing with the casein blocker solution, AbC-MBs were incubated with the samples supplemented with the HRP-labelled detector antibody (HRP-AbD), the target protein being sandwiched between the AbC immobilized on the MBs and the HRP-AbD. The as-prepared MBs, bearing the sandwich immunocomplexes, were captured magnetically on the working electrode surface by placing the SPCEs on a custom-fabricated magnetic holding block, and the extent of the biorecognition event was monitored by amperometry in stirred solutions of the reduction current generated upon H_2_O_2_ addition in the presence of HQ. 

### 3.1. Optimization of Experimental Variables

In order to evaluate non-specific binding of the antigen and/or detector antibody on the activated MBs’ surface, we compared the amperometric responses obtained for 0.0 and 100 ng·mL^−1^ human p53 standards with and without AbC immobilized on the MBs. Results showed negligible non-specific adsorption of the HRP-AbD, but large non-specific adsorption of the target protein at AbC-free MBs. In order to minimize these non-specific signals, 1 M ethanolamine and commercial casein solution blocking agents were tested, as well as different blocking protocols (only one blocking process either with a 1 M ethanolamine solution or with the commercial casein solution, or two successive blocking steps, first with 1 M ethanolamine and then with the casein solution). The resulting amperometric measurements (data not shown) showed that the target protein was immobilized to the MBs through the AbC only when two successive blocking steps with 1 M ethanolamine and the commercial blocker casein solution were performed after the AbC immobilization onto the MBs. All other blocking protocols did not avoid significant non-specific adsorption of the human p53 protein to the unmodified MBs, thus preventing proper implementation of the sandwich configuration. Once the non-specific adsorptions were minimized, all of the experimental variables involved in the immunosensor preparation were optimized. The criterion of selection adopted for optimization was the largest current ratio between the measurements for 100 (signal, S) and 0.0 (blank, B) ng·mL^−1^ human p53 standard solutions (signal-to-blank, S/B, ratio) obtained at −0.20 V (vs. the Ag pseudo-reference electrode). This detection potential was previously optimized for the HRP/HQ/H_2_O_2_ system [15]. Table 1 summarizes all the optimized variables, the corresponding ranges into which they were checked and the values selected for the preparation of the MBs-based immunosensor. 

**Figure 2:**
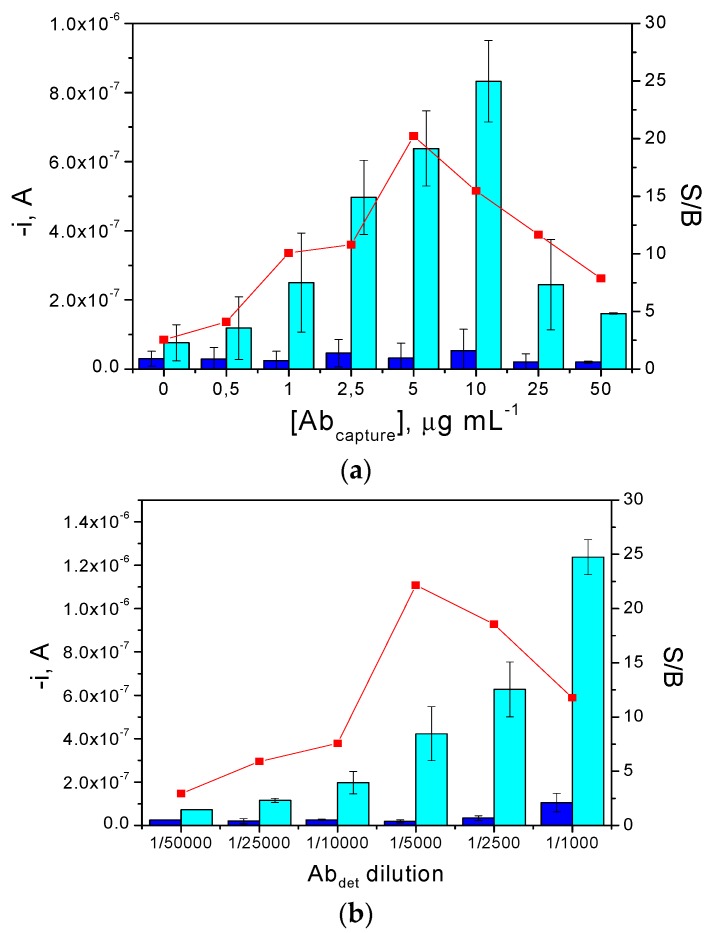
Dependence of the S/B current ratio (red ▪) with the AbC loading (**a**) and AbD dilution factor used to perform the immunoassay (**b**). Amperometric responses were measured for 0 (dark blue bars) and 100 ng·mL^−1^ of human p53 standard (light blue bars). Error bars were estimated as triple that of the standard deviation (*n* = 3).

As examples, results obtained in the optimization of the AbC loading and the dilution applied to the commercial AbD solution, are provided in Figure 2a,b, respectively. As expected, while there are no significant differences between the B signals (obtained in the absence of target protein), the current measured at −0.20 V for 100 ng·mL^−1^ human p53 increased significantly with the AbC loading (Figure 2a) up to 50 µg/ mL, and then decreased drastically, most likely due to the steric hindrance of the antigen when large amounts of the capture antibody are immobilized [18]. Regarding the dilution applied to the AbD solution (Figure 2b), the currents measured both in the presence and in absence of p53 protein increased with the AbD concentration. This behavior was attributed to an increase in the non-specific adsorption of this antibody. A 1/5000 dilution factor, where the highest S/B ratio was observed, was chosen for further experiments.

Regarding the number of steps involved in the immunoassay working protocol, two different procedures were checked: (1) target protein capture and sandwiching with the labeled detector antibody were carried out in a single step by 30 min incubation of the AbC-MBs in a mixture solution containing human p53 standard and HRP-AbD; (2) two sequential steps involving 30 min incubation of the AbC-MBs in the p53 protein solution, followed by another 30 min incubation in the HRP-AbD solution. Results (not shown) indicated a slightly higher S/B current ratio when only one single incubation step was used which, in addition, considerably reduced the total assay time. This behavior has been also observed for other sandwich immunoassays [19,20] and can be attributed to a higher efficiency of the immune and labeling reactions due to the lower steric hindrance occurring when the target antigen and the detector antibody are free in homogeneous solution. Accordingly, the shorter one-step protocol was selected for further studies. 

### 3.2. Analytical Characteristics of the Immunosensor

The reproducibility of the amperometric responses obtained for 50 ng·mL^−1^ human p53 standards was evaluated with ten different immunosensors. A relative standard deviation (RSD) value of 3.3% was calculated demonstrating the reliability of the whole procedure including both the immunosensor fabrication (MBs modification and magnetic capture on the SPCE surface) and the amperometric transduction. 

**Figure 3:**
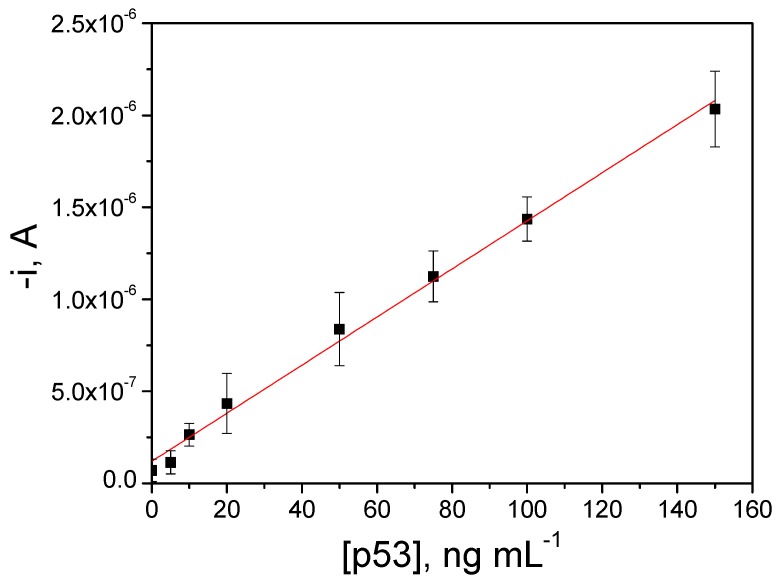
Calibration plot constructed with human p53 standards. Error bars were estimated as triple that of the standard deviation (*n* = 3).

The calibration plot constructed for human p53 standards under the selected experimental conditions (Figure 3) exhibited a linear range (r = 0.998) between the measured current and the p53 concentration from 5–150 ng·mL^−1^, with a slope value of (1.3 ± 0.1) × 10^−8^ A·mL·ng^−1^ and an intercept of (1.4 ± 0.8) × 10^−7^ A. It is important to mention that, despite using a one-step sandwich immunoassay, no induction of any hook effect was observed at the tested concentration levels. This effect would only be expected for much larger concentrations than those usually considered as cut-off values in clinical samples for p53 overexpression in cancer ailments, thus ensuring the absence of potential misdiagnosis due to falsely decreased results. Detection (LOD) and quantification (LQ) limits, estimated according to the 3 × s_b_/m and 10 × s_b_/m criteria, respectively, where m is the slope of the linear calibration plot, and s_b_ is the standard deviation of 10 amperometric signals measured in the absence of the target, were 1.29 and 4.31 ng·mL^−1^, respectively. 

The storage stability of AbC-MBs conjugates, once the ethanolamine blocking step was performed, was evaluated by keeping them at 4 °C in filtered PBS. Each working day, a few of the prepared conjugates were incubated with fresh solutions of HRP-AbD supplemented with 0 or 50 ng·mL^−1^ human p53 standard, according to the protocols described in Section 2.3 and Section 2.4. No significant differences in the measured current S/B ratio were apparent for a period of seven days, indicating acceptable storage stability of the AbC-MBs bioconjugates, which can be prepared in the lab and stored until used for point-of-care testing at bed side. 

Table 2 compares the main features of the developed immunosensor with other electrochemical immunosensors described so far for the determination of both phosphorylated and non-phosphorylated human p53. Although the LOD achieved is not as low as those reported by other authors, all of them using nanomaterials-based amplification systems, this immunosensor offered a sensitivity adequate for the determination of the target protein in cell lysates without any amplification step as it will be demonstrated below. It is worth remarking, also, of the short time required to prepare the immunosensor, in comparison with that required with the other methodologies. Moreover, the 45 min assay time required with the developed inmunosensor is 2–4 times shorter than that of other approaches free of immunoreactions acceleration strategies [7,8,9]. Furthermore, the inherent simplicity of the method reported here can be also claimed as an important practical advantage versus all of other approaches reported so far, which require multiple reagents and complex and time-consuming substrate/nanomaterials modification protocols, thus making them difficult to be considered as suitable tools for the development of user-friendly devices for on-site determination of this relevant biomarker.

### 3.3. Selectivity of the Magnetoimmunosensor

**Figure 4:**
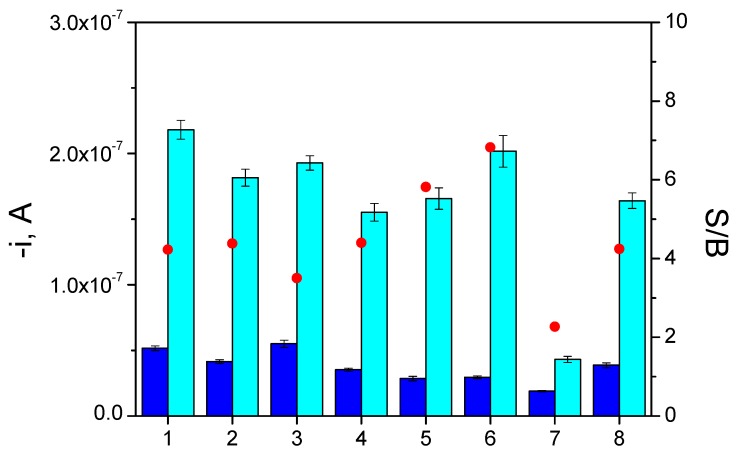
Immunosensor selectivity towards p53 protein. Current values measured for 0.0 (dark blue bars) and 10.0 (light blue bars) ng·mL^−1^ human p53 standard in the absence (1) and in the presence of: 10.0 ng·mL^−1^ TNFα (2); 5.0 ng·mL^−1^ ErbB2 (3); 5.0 ng·mL^−1^ ERα (4); 5.0 ng·mL^−1^ PR (5); 5.0 mg·mL^−1^ BSA (6); 1.0 mg·mL^−1^ human IgG (7) and 0.1 mg·mL^−1^ human IgG (8). Supporting electrolyte, 0.05 M sodium phosphate solution, pH 6.0; E_app_ = −0.20 V vs. the Ag pseudo-reference electrode. Other conditions are as described in Table 1 (selected values column). S/B ratio (red •) are those obtained for each experimental point. Error bars estimated as triple that of the standard deviation (*n* = 3).

The selectivity of the developed immunosensor was evaluated towards other cancer biomarkers and against non-target proteins which can coexist with p53 protein in human serum. These tests were performed by comparing the current values measured with the immunosensor for 0.0 and 10.0 ng·mL^−1^ human p53 standards both in the absence and in the presence of these potential interfering compounds at a similar concentration to that of the target biomarker or at their usual or higher protein concentrations in serum samples. Figure 4 shows that the reliable determination of 10 ng·mL^−1^ of the target protein was possible in the presence of all the other non-target proteins at the assayed concentration levels except for human IgG. The significant interference observed in the presence of 1.0 mg·mL^−1^ human IgG, already reported by other authors [20,21,22] in sandwich immunoassays using mouse monoclonal antibodies, is attributed to the presence of human anti-mouse antibodies (HAMAs) which show specificity for mouse immunoglobulins and can cross-link the capture and labeled antibodies in the absence of the analyte. Although no significant interference was observed in the presence of human IgG only at a 0.1 mg·mL^−1^ concentration level (see bars 8 in Figure 4), it is important to mention that this interference will not pose any problem in the analysis of cell lysates without endogenous content of this non-target protein.

### 3.4. Determination of Human p53 in Cell Lysates

The real usefulness of the developed methodology was evaluated by determining the endogenous content of the target protein in cell lysates expressing different p53 levels. Since no statistically significant differences were observed between the slope value of the calibration plot constructed with human p53 standards and the slope values of the calibration graphs recorded for cell lysates assayed (once they were adequately diluted with blocker casein solution and spiked with growing amounts of a standard human p53 solution up to 100.0 ng·mL^−1^ and supplemented with 5000 times diluted HRP-AbD), we concluded that no significant matrix effects were apparent in these complex samples. Therefore, the endogenous concentration of human p53 in cell lysates was quantified in a straightforward manner by interpolating the amperometric responses obtained with the diluted samples (up to a final lysate amount of 2.0 µg) into the calibration plot prepared with human p53 standards (Figure 3).

**Figure 5:**
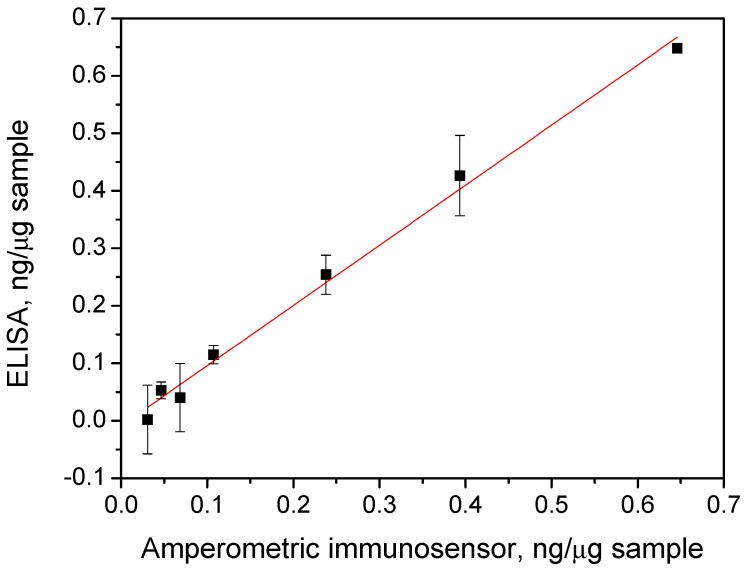
Comparison of the results obtained with the magnetoimmunosensor and the ELISA methodologies. Dots in the graph correspond to (from left to right) BxPc3, MCF-7, KM12SM, SW620, KM12C, MDA-MB-436, and SW480, respectively. Error bars are estimated as a triple that of the standard deviation (*n* = 3).

The obtained results were compared with those provided by a commercial ELISA kit. A paired samples *t*-test demonstrated that no significant differences (α = 0.05) existed between the results found by both methods (*p*-value = 0.50). It is important to note the reliability of the approach just after a simple dilution with blocker casein solution despite the complexity of samples. The plot of the mean contents obtained with the ELISA kit versus those provided by the magnetoimmunosensor (Figure 5) resulted in a linear least-squares regression graph (r = 0.997) with a slope value of (1.04 ± 0.04) and an intercept of (−0.01 ± 0.01). As can be observed, the correlation found was highly satisfactory since the confidence intervals (at a significance level of α = 0.05) for the slope and intercept included the unit and the zero values, respectively, indicating that the methodology involving the use of the magnetoimmunosensor exhibited no systematic errors and can be successfully used for the reliable determination of p53 protein in cell lysates.

These results pointed out that, despite the LOD achieved with the developed immunosensor being higher than that claimed for the ELISA spectrophotometric kit using the same immunoreagents (1.29 ng·mL^−1^ vs. 100 pg·mL^−1^), the sensitivity of the developed approach is suitable for real practice. Moreover, the use of the MBs-based immunosensor allowed the analysis to be made in approximately seven times shorter a time than the ELISA method (45 vs. 300 min once the AbC-MBs and AbC-plate were prepared and blocked, respectively), and in a simplified analytical procedure requiring only one incubation step with the sample solution supplemented with the HRP-AbD. Therefore, the significantly shorter assay time, the inherent simplicity, and the involvement of portable and cost-effective instrumentation of the approach reported here can be claimed as important practical advantages over commercial ELISAs, which use tedious, time-consuming multistage processes and expensive detection instrumentation, making them difficult to implement as a tool for the development of user-friendly devices to perform routine and decentralized analysis. Although the developed methodology applicability has only been tested with cell lysates, it may also be applied to the determination of the target protein in other samples with clinical relevance, such as tissues’ biopsies or exosomes.

## 4. Conclusions

A simple electrochemical methodology for the determination of human p53 protein, based on the use of immune-MBs as selective capture microcarriers and amperometric detection on SPCEs, has been developed for the first time and applied to the analysis of cell lysates. Determination of p53 protein is carried out sandwiching it between specific capture antibodies covalently attached to previously-activated carboxylated MBs and HRP-labeled detector antibodies. The concentration of the target protein is related with the amperometric signal obtained (at −0.20 V vs. a Ag pseudoreference electrode) after magnetic capturing of the resulting modified MBs on the surface of a SPCE upon the addition of H_2_O_2_ and in the presence of HQ. The developed bioplatforms, with 7-day storage stability, allowed the selective and sensitive determination of the target protein (LOD of 1.2 ng·mL^−1^ for human p53 standards) without any signal amplification and successful applicability to the analysis of cell lysates. Moreover, the rapid, simple, portable, and cheap operation make this immunosensor a very attractive alternative to commonly-used ELISAs for the development of automated devices for on-site and routine determinations. 

## Figures and Tables

**Table 1:** Different experimental variables optimized to develop the amperometric human 53 immunosensor.

Variable	Tested Range	Selected Value
V_HOOC-MBs_, µL	1–6	3
[AbC], µg·mL^−1^	0.0–50.0	5.0
t_incubation AbC_, min	0–90	30
HRP-AbD dilution factor	1/50,000–1/1000	1/5000
t_incubation HRP-AbC_, min	0–60	30
t_blocking ethanolamine_, min	0–60	15
t_blocking commercial blocker casein solution_, min	0–60	15
Steps number	1–2	1

**Table 2:** Characteristics of electrochemical biosensors for the detection of p53 protein.

Working Electrode	Detector Antibody Labelling	Transduction Technique	Sample	Concentration Range	LOD	Preparation Time *	Assay Time **	Reference
Graphene-chitosan-SPCE	HRP-streptavidin-biotin	DPV	-	0.2–10 ng·mL^−1^	0.1 ng·mL^−1^	~4 h	~2 h	[9]
AuNPs-SPCE	HRP-GO	SWV	Spiked human plasma	0.02–2 nM	0.01 nM	>4 h	>2 h	[7]
NHS-SPGE	HRP-Au nanorods	SWV	-	0.01–20 nM (phospho-p53^392^) 0.05–20 nM (phospho-p53^15^) 0.1–50 nM (phospho-p53^46^) 0.05–20 nM (total p53)	5 pM (phospho-p53^392^) 20 pM (phospho-p53^15^) 30 pM (phospho-p53^46^) 10 pM (total p53)	>4 h	~5 min	[8]
Bi-SPCE	PCN-NS	SWV	Human serum	0.02–20 ng·mL^−1^	0.01 ng·mL^−1^	~3 h	~1.5 h	[14]
Thiolated GO-streptavidin-AuNPs-GCE	Avidin-biotin-HRP	DPV	Cell lysates. Normal and cancerous human skin fibroblast cells	0.2–2 pM	30 fM	~20 h	~3 h	[4]
SPCE	HRP	Amperometry	Cell lysates	5–150 ng·mL^−1^ (69 nM–2.1 µM)	1.29 ng·mL^−1^ (18 nM)	~2 h	~45 min	This work

* Estimated time to prepare the immunosensor (fabrication of nanostructures and/or antibodies labelling procedures not included); ** Estimated time from the application of the sample to the system till the signal measurement. AuNPs, gold nanoparticles; DPV, differential pulse voltammetry; GCE, glassy carbon electrode; GO, graphene oxide; HRP, horseradish peroxidase; NHS, N-hydroxysuccinimide-activated hexa(ethylene glycol) undecane thiol; NS, carbon nanospheres; PCN, protein cage nanoparticles; SPCE, screen-printed carbon electrode; SPGE, screen-printed gold electrode; SWV, square wave voltammetry.
